# Regulation of the boron transporter EgBor1 in *Eucalyptus globulus*: a plausible model

**DOI:** 10.1186/1753-6561-5-S7-P91

**Published:** 2011-09-13

**Authors:** Hans Pieringer, Fabiola Baltierra, Maria Cecilia Gamboa, Erwin Krauskopf

**Affiliations:** 1 Fundacion Ciencia para la Vida, Santiago, Chile; 2 Facultad de Ciencias Biologicas, Universidad Andres Bello, Santiago, Chile

## 

Boron is a micronutrient that plays an important role in plant cell wall biosynthesis. Nevertheless, an excess of boron in the soil causes severe damage to the respiratory tissue of the plant. In *Arabidopsis thaliana*, the *Atbor1* gene encodes a boron transporter that distributes this element throughout the plant.

A cDNA sequence encoding a *bor1* transporter was isolated from a *Eucalyptus globulus* cDNA library. This sequence contains several stop codons within the coding region. Initial bioinformatic analyses suggest that this interruption corresponds to an intron that may generate a truncated protein.

*Egbor1*was overexpressed in *Saccharomyces cereviseae* to assess whether it was capable of restoring the phenotype of a mutant strain that lacks the boron transporter. It was also overexpressed in a wild type strain as a control. In both cases a significant increase in boron tolerance was observed, suggesting that the encoded transporter is functional. Subsequently, a western blot analysis showed that the expressed transporter corresponds to the product of the full-length protein, rather than the truncated protein.

Additional bioinformatic analyses showed that the intron presents several regulatory elements, therefore it may function as a promoter for a small protein encoded in the 3’ region of *Egbor1*. Transient expression of GUS under the control of the intron sequence proved its capability to activate gene expression. Thus, we have identified three ORFs in the *Egbor1* sequence: a full length protein encoded by the spliced mRNA named fragment C, and two smaller proteins encoded by the 5’ and the 3’ regions of the non spliced mRNA named fragments A and B.

We are currently challenging the yeast mutant strain with all the putative encoded proteins to understand their specific role in boron transport. Funded by PFB-016 and DI-UNAB.

